# Supplementary figures and images for: Structural evolution of nitrogenase over 3 billion years
Source: eLife. 2025 Sep 11;14:RP105613. doi: 10.7554/eLife.105613 (PMC12425478; doi:10.7554/eLife.105613)

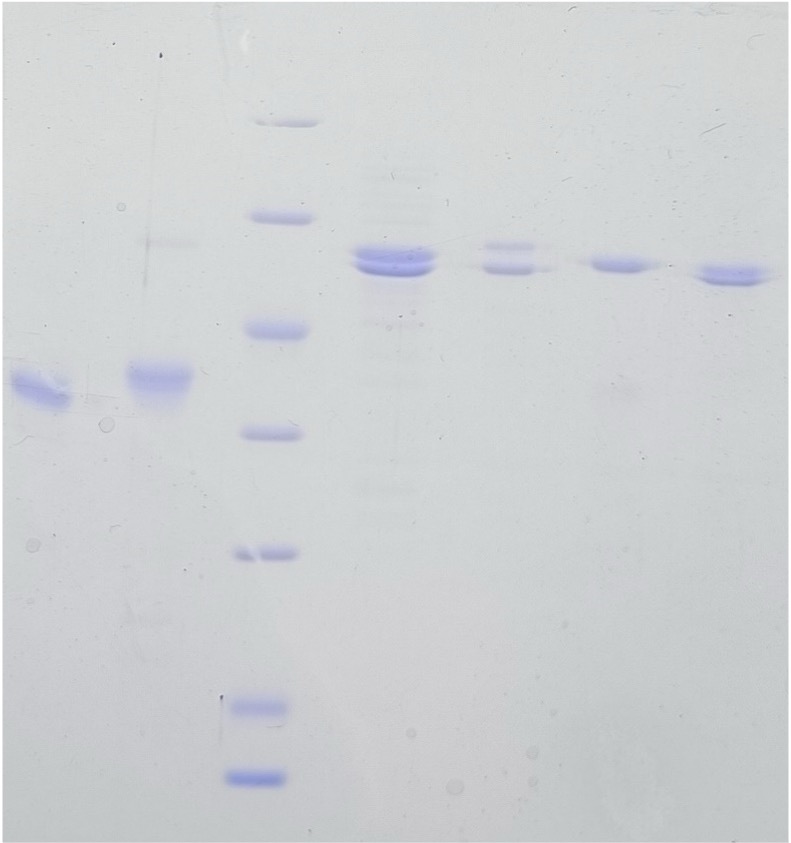

Supplement: Figure 5—figure supplement 1—source data 1. [file elife-105613-fig5-figsupp1-data1.jpg]

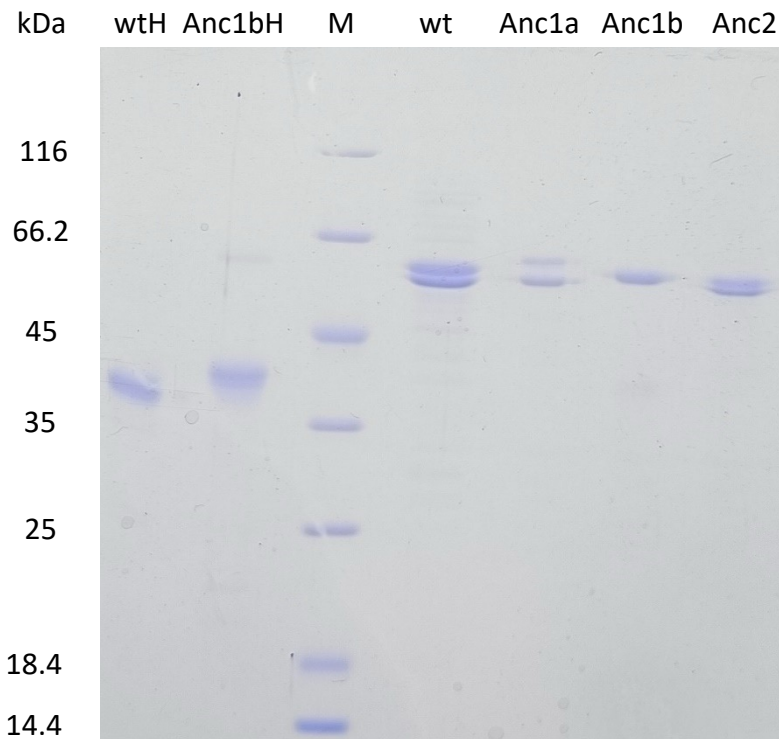

Supplement: Figure 5—figure supplement 1—source data 2. [file elife-105613-fig5-figsupp1-data2.pdf]
