# Supplementary material for: Structural evolution of nitrogenase over 3 billion years
Source: eLife. 2025 Sep 11;14:RP105613. doi: 10.7554/eLife.105613 (PMC12425478; doi:10.7554/eLife.105613)
Supplement: Supplementary file 1. — H, D, K, and G refer to the respective H, D, K, and G-subunits of nitrogenase. The numbering refers to the oligomeric state for each of the subunit in the complex. [file elife-105613-supp1.docx]

***Supplementary File 1*.** Crystallographic or cryo-EM nitrogenase structures presently available in the Protein Data Bank (<https://www.rcsb.org/>), accessed September 2023. H,D,K and G refer to the respective H, D, K and G-subunits of nitrogenase. The numbering refers to the oligomeric state for each of the subunit in the complex.

| **PDB ID** | **Organism** | Resolution | Year | Type | Stoichiometry |
| --- | --- | --- | --- | --- | --- |
| 8BOQ | *Azotobacter vinelandii* | 1.55 | 2023 | Anf | D2G2K2 |
| 8DFD | *Azotobacter vinelandii* | 2.12 | 2023 | Nif | D2H4K2 |
| 8DBY | *Azotobacter vinelandii* | 2.26 | 2023 | Nif | D2K2 |
| 8DFC | *Azotobacter vinelandii* | 2.48 | 2023 | Nif | D2H2K2 |
| 1NIP | *Azotobacter vinelandii* | 2.90 | 1992 | Nif | H2 |
| 2MIN | *Azotobacter vinelandii* | 2.03 | 1997 | Nif | D2K2 |
| 3MIN | *Azotobacter vinelandii* | 2.03 | 1997 | Nif | D2K2 |
| 1N2C | *Azotobacter vinelandii* | 3.00 | 1997 | Nif | D2H4K2 |
| 2NIP | *Azotobacter vinelandii* | 2.20 | 1998 | Nif | H2 |
| 1FP6 | *Azotobacter vinelandii* | 2.15 | 2000 | Nif | H4 |
| 1DE0 | *Azotobacter vinelandii* | 2.40 | 2000 | Nif | H2 |
| 1G5P | *Azotobacter vinelandii* | 2.20 | 2001 | Nif | H2 |
| 1G20 | *Azotobacter vinelandii* | 2.20 | 2001 | Nif | D2H4K2 |
| 1G1M | *Azotobacter vinelandii* | 2.25 | 2001 | Nif | H2 |
| 1FP4 | *Azotobacter vinelandii* | 2.50 | 2001 | Nif | D2K2 |
| 1G21 | *Azotobacter vinelandii* | 3.00 | 2001 | Nif | D2H4K2 |
| 1M1N | *Azotobacter vinelandii* | 1.16 | 2002 | Nif | D4K4 |
| 1L5H | *Azotobacter vinelandii* | 2.30 | 2002 | Nif | D1K1 |
| 1M34 | *Azotobacter vinelandii* | 2.30 | 2002 | Nif | D4H8K4 |
| 1M1Y | *Azotobacter vinelandii* | 3.20 | 2002 | Nif | D4H8K4 |
| 1RW4 | *Azotobacter vinelandii* | 2.50 | 2004 | Nif | H1 |
| 1XD8 | *Azotobacter vinelandii* | 2.70 | 2004 | Nif | H2 |
| 1XD9 | *Azotobacter vinelandii* | 2.80 | 2004 | Nif | H2 |
| 1XDB | *Azotobacter vinelandii* | 2.80 | 2004 | Nif | H2 |
| 1XCP | *Azotobacter vinelandii* | 3.20 | 2004 | Nif | H4 |
| 2AFH | *Azotobacter vinelandii* | 2.10 | 2005 | Nif | D2H2K2 |
| 4WZB | *Azotobacter vinelandii* | 2.30 | 2005 | Nif | D2H4K2 |
| 2AFI | *Azotobacter vinelandii* | 3.10 | 2005 | Nif | D4H8K4 |
| 2C8V | *Azotobacter vinelandii* | 2.50 | 2006 | Nif | H1 |
| 3K1A | *Azotobacter vinelandii* | 2.23 | 2010 | Nif | D2K2 |
| 3U7Q | *Azotobacter vinelandii* | 1.00 | 2011 | Nif | D2K2 |
| 4TKU | *Azotobacter vinelandii* | 1.43 | 2014 | Nif | D2K2 |
| 4TKV | *Azotobacter vinelandii* | 1.50 | 2014 | Nif | D2K2 |
| 4ND8 | *Azotobacter vinelandii* | 2.00 | 2014 | Nif | D2K2 |
| 5BVH | *Azotobacter vinelandii* | 1.53 | 2015 | Nif | D2K2 |
| 5BVG | *Azotobacter vinelandii* | 1.60 | 2015 | Nif | D2K2 |
| 5CX1 | *Azotobacter vinelandii* | 1.75 | 2015 | Nif | D8K8 |
| 4WZA | *Azotobacter vinelandii* | 1.90 | 2015 | Nif | D2H4K2 |
| 4XPI | *Azotobacter vinelandii* | 1.97 | 2015 | Nif | D2K2 |
| 4WNA | *Azotobacter vinelandii* | 2.00 | 2015 | Nif | D2K2 |
| 6BBL | *Azotobacter vinelandii* | 1.68 | 2017 | Nif | D2K2 |
| 5VQ4 | *Azotobacter vinelandii* | 2.30 | 2017 | Nif | D2K2 |
| 6CDK | *Azotobacter vinelandii* | 2.10 | 2018 | Nif | D2K2 |
| 6N4L | *Azotobacter vinelandii* | 1.13 | 2019 | Nif | H1 |
| 6O7M | *Azotobacter vinelandii* | 1.40 | 2019 | Nif | D2K2 |
| 6N4M | *Azotobacter vinelandii* | 1.58 | 2019 | Nif | H1 |
| 6O0B | *Azotobacter vinelandii* | 1.60 | 2019 | Nif | H2 |
| 6OP3 | *Azotobacter vinelandii* | 1.60 | 2019 | Nif | D2K2 |
| 6O7P | *Azotobacter vinelandii* | 1.70 | 2019 | Nif | D2K2 |
| 6OP1 | *Azotobacter vinelandii* | 1.70 | 2019 | Nif | D2K2 |
| 6O7N | *Azotobacter vinelandii* | 1.75 | 2019 | Nif | D2K2 |
| 6N4K | *Azotobacter vinelandii* | 1.76 | 2019 | Nif | H2 |
| 6O7O | *Azotobacter vinelandii* | 1.89 | 2019 | Nif | D2K2 |
| 6OP2 | *Azotobacter vinelandii* | 1.90 | 2019 | Nif | D2K2 |
| 6N4J | *Azotobacter vinelandii* | 1.95 | 2019 | Nif | H2 |
| 6O7Q | *Azotobacter vinelandii* | 2.00 | 2019 | Nif | D2K2 |
| 6O7L | *Azotobacter vinelandii* | 2.26 | 2019 | Nif | D2K2 |
| 6O7S | *Azotobacter vinelandii* | 2.27 | 2019 | Nif | D2K2 |
| 6O7R | *Azotobacter vinelandii* | 2.27 | 2019 | Nif | D2K2 |
| 6OP4 | *Azotobacter vinelandii* | 2.30 | 2019 | Nif | D2K2 |
| 6VXT | *Azotobacter vinelandii* | 1.74 | 2020 | Nif | D2K2 |
| 6UG0 | *Azotobacter vinelandii* | 1.83 | 2020 | Nif | D2K2 |
| 7JRF | *Azotobacter vinelandii* | 1.33 | 2021 | Nif | D2K2 |
| 7TPW | *Azotobacter vinelandii* | 1.18 | 2022 | Nif | H1 |
| 7TPO | *Azotobacter vinelandii* | 1.35 | 2022 | Nif | H1 |
| 7TPX | *Azotobacter vinelandii* | 1.35 | 2022 | Nif | H1 |
| 7TPN | *Azotobacter vinelandii* | 1.38 | 2022 | Nif | H1 |
| 7TNE | *Azotobacter vinelandii* | 1.39 | 2022 | Nif | H1 |
| 7TQF | *Azotobacter vinelandii* | 1.45 | 2022 | Nif | H1 |
| 7TQI | *Azotobacter vinelandii* | 1.47 | 2022 | Nif | H1 |
| 7TPY | *Azotobacter vinelandii* | 1.48 | 2022 | Nif | H1 |
| 7TQJ | *Azotobacter vinelandii* | 1.48 | 2022 | Nif | H1 |
| 7TQK | *Azotobacter vinelandii* | 1.48 | 2022 | Nif | H1 |
| 7TPV | *Azotobacter vinelandii* | 1.49 | 2022 | Nif | H1 |
| 7TQH | *Azotobacter vinelandii* | 1.49 | 2022 | Nif | H1 |
| 7T4H | *Azotobacter vinelandii* | 1.51 | 2022 | Nif | H1 |
| 7TQC | *Azotobacter vinelandii* | 1.53 | 2022 | Nif | H1 |
| 7TQE | *Azotobacter vinelandii* | 1.59 | 2022 | Nif | H1 |
| 7TQ9 | *Azotobacter vinelandii* | 1.60 | 2022 | Nif | H1 |
| 7TPZ | *Azotobacter vinelandii* | 1.71 | 2022 | Nif | H1 |
| 7TQ0 | *Azotobacter vinelandii* | 1.81 | 2022 | Nif | H1 |
| 8DBX | *Azotobacter vinelandii* | 1.92 | 2023 | Nif | D2K2 |
| 8CRS | *Azotobacter vinelandii* | 2.04 | 2023 | Nif | D2K2 |
| 8ENM | *Azotobacter vinelandii* | 2.14 | 2023 | Nif | D2K2 |
| 8ENL | *Azotobacter vinelandii* | 2.37 | 2023 | Nif | D2K2 |
| 8BTS | *Azotobacter vinelandii* | 3.03 | 2023 | Nif | D4K4 |
| 5N6Y | *Azotobacter vinelandii* | 1.35 | 2017 | Vnf | D2G2K2 |
| 6FEA | *Azotobacter vinelandii* | 1.20 | 2018 | Vnf | D2G2K2 |
| 6Q93 | *Azotobacter vinelandii* | 2.20 | 2018 | Vnf | H8 |
| 7ADR | *Azotobacter vinelandii* | 1.00 | 2020 | Vnf | D2G2K2 |
| 7ADY | *Azotobacter vinelandii* | 1.05 | 2020 | Vnf | D2G2K2 |
| 7AIZ | *Azotobacter vinelandii* | 1.05 | 2021 | Vnf | D2G2K2 |
| 1MIO | *Clostridium pasteurianum* | 3.00 | 1993 | Nif | D2K2 |
| 1CP2 | *Clostridium pasteurianum* | 1.93 | 1998 | Nif | H2 |
| 4WN9 | *Clostridium pasteurianum* | 1.90 | 2015 | Nif | D2K2 |
| 5VQ3 | *Clostridium pasteurianum* | 1.72 | 2017 | Nif | D2K2 |
| 5VPW | *Clostridium pasteurianum* | 1.85 | 2017 | Nif | D2K2 |
| 5KOH | *Gluconacetobacter*  *diazotrophicus* PA1 5 | 1.83 | 2016 | Nif | D2K2 |
| 5KOJ | *Gluconacetobacter*  *diazotrophicus* PA1 5 | 2.59 | 2016 | Nif | D2K2 |
| 1QH8 | *Klebsiella pneumoniae* | 1.60 | 1999 | Nif | D2K2 |
| 1QH1 | *Klebsiella pneumoniae* | 1.60 | 1999 | Nif | D2K2 |
| 1QGU | *Klebsiella pneumoniae* | 1.60 | 1999 | Nif | D2K2 |
| 1H1L | *Klebsiella pneumoniae* | 1.90 | 2002 | Nif | D2K2 |
| 6NZJ | *Methanosarcina*  *acetivorans* C2A | 2.40 | 2019 | Nif | H2 |
