# Supplementary material for: Structural evolution of nitrogenase over 3 billion years
Source: eLife. 2025 Sep 11;14:RP105613. doi: 10.7554/eLife.105613 (PMC12425478; doi:10.7554/eLife.105613)
Supplement: Supplementary file 2. [file elife-105613-supp2.docx]

**Supplementary File 2**: Data collection and refinement statistics for Anc2

| **Data collection** |  |
| --- | --- |
| Wavelength (Å) | 1.000 |
| Spherical resolution (Å) | 110.49-1.816 (2.013-1.816) |
| Limiting resolution (Å) along |  |
| a* | 2.417 |
| b* | 1.984 |
| c* | 1.816 |
| Space group | P 2_1_2_1_2_1_ |
| Unit cell | 74.936, 130.133, 209.137, 90, 90, 90, |
| Total reflections | 1683610 (81981) |
| Unique reflections | 121997 (6101) |
| Multiplicity | 13.80 (13.4) |
| Completeness, spherical (%) | 66.51 (12.6) |
| Completeness, ellipsoidal (%) | 95.0 (70.2) |
| Mean I/sigma(I) | 10.0 (1.6) |
| Wilson B-factor | 21.85 |
| R-merge (Weiss and Hilgenfeld,1997) | 0.202 (1.696) |
| R-meas | 0.210 (1.772) |
| R-pim (Weiss and Hilgenfeld, 1997) | 0.056 (0.478) |
| CC_1/2_ (Karplus and Diederichs, 2012) | 0.996 (0.698) |
| **Refinement** |  |
| R-work | 0.1864 |
| R-free | 0.2305 |
| RMS(bonds) | 0.024 |
| RMS(angles) | 1.68 |
| Ramachandran favored (%) | 96.44 |
| Ramachandran allowed (%) | 3.36 |
| Ramachandran outliers (%) | 0.20 |
| Rotamer outliers (%) | 1.62 |
| Clashscore | 3.88 |
| Average B-factor | 27.72 |
| ..macromolecules | 27.57 |
| ..ligands | 23.48 |
| ..solvent | 29.97 |

Statistics for the highest-resolution shell are shown in parentheses.

**References**:

Weiss MS, Hilgenfeld R. 1997. On the use of the merging R factor as a quality indicator for X-ray data. *J Appl Crystallogr* **30**:203–205. doi:10.1107/s0021889897003907

Karplus PA, Diederichs K. 2012. Linking Crystallographic Model and Data Quality. *Science* **336**:1030–1033. doi:10.1126/science.1218231
