# Supplementary material for: Structural evolution of nitrogenase over 3 billion years
Source: eLife. 2025 Sep 11;14:RP105613. doi: 10.7554/eLife.105613 (PMC12425478; doi:10.7554/eLife.105613)
Supplement: Supplementary file 3. [file elife-105613-supp3.docx]

**Supplementary File 3:** Data collection and refinement statistics for Anc1A

| **Data collection** |  |
| --- | --- |
| Wavelength (Å) | 0.8856 |
| Spherical resolution (Å) | 19.791-2.658 (2.912-2.658) |
| Limiting resolution (Å) along |  |
| a* | 2.934 |
| b* | 2.656 |
| c* | 2.913 |
| Space group | P 2_1_2_1_2_1_ |
| Unit cell | 70.29, 138.61, 208.58, 90.0, 90.0, 90.0 |
| Total reflections | 595126 (29588) |
| Unique reflections | 46401 (2321) |
| Multiplicity | 12.8 (12.7) |
| Completeness, spherical (%) | 78.07 (16.5) |
| Completeness, ellipsoidal (%) | 93.7 (51.8) |
| Mean I/sigma(I) | 7.1 (1.4) |
| Wilson B-factor | 39.22 |
| R-merge (Weiss and Hilgenfeld,1997) | 0.368 (2.046) |
| R-meas | 0.384 (2.131) |
| R-pim (Weiss and Hilgenfeld,1997) | 0.106 (0.593) |
| CC_1/2_ (Karplus and Diederichs, 2012) | 0.989 (0.425) |
| **Refinement** |  |
| R-work | 0.1796 |
| R-free | 0.2220 |
| RMS(bonds) | 0.022 |
| RMS(angles) | 1.44 |
| Ramachandran favored (%) | 95.53 |
| Ramachandran allowed (%) | 4.47 |
| Ramachandran outliers (%) | 0.00 |
| Rotamer outliers (%) | 5.57 |
| Clashscore | 3.95 |
| Average B-factor | 42.75 |
| ..macromolecules | 42.91 |
| ..ligands | 42.28 |
| solvent | 23.71 |

Statistics for the highest-resolution shell are shown in parentheses.

**References**:

Weiss MS, Hilgenfeld R. 1997. On the use of the merging R factor as a quality indicator for X-ray data. *J Appl Crystallogr* **30**:203–205. doi:10.1107/s0021889897003907

Karplus PA, Diederichs K. 2012. Linking Crystallographic Model and Data Quality. *Science* **336**:1030–1033. doi:10.1126/science.1218231
