# Supplementary material for: Structural evolution of nitrogenase over 3 billion years
Source: eLife. 2025 Sep 11;14:RP105613. doi: 10.7554/eLife.105613 (PMC12425478; doi:10.7554/eLife.105613)
Supplement: Supplementary file 4. [file elife-105613-supp4.docx]

**Supplementary File 4**: Data collection and refinement statistics for Anc1B HH

| **Data collection** |  |
| --- | --- |
| Wavelength (Å) | 0.8856 |
| Spherical resolution (Å) | 69.75 - 2.434 (2.521 - 2.434) |
| Limiting resolution (Å) along |  |
| a* | 2.434 |
| b* | 2.468 |
| c* | 2.828 |
| Space group | C 12_1_ |
| Unit cell | 123.35, 109.78, 95.21, 90.0, 118.6, 90.0 |
| Total reflections | 45985 (2294) |
| Unique reflections | 26823 (1341) |
| Multiplicity | 1.7 (1.7) |
| Mean I/sigma(I) | 2.74 (1.52) |
| Wilson B-factor | 28.93 |
| R-merge (Weiss and Hilgenfeld,1997) | 0.096 (0.321) |
| R-meas | 0.132 (0.447) |
| R-pim (Weiss and Hilgenfeld,1997) | 0.09 (0.310) |
| CC_1/2_ (Karplus and Diederichs, 2012) | 0.98 (0.716) |
| **Refinement** |  |
| R-work | 0.2023 |
| R-free | 0.2722 |
| RMS(bonds) | 0.008 |
| RMS(angles) | 1.49 |
| Ramachandran favored (%) | 95.72 |
| Ramachandran allowed (%) | 3.75 |
| Ramachandran outliers (%) | 0.52 |
| Rotamer outliers (%) | 9.31 |
| Clashscore | 7.19 |
| Average B-factor | 29.54 |
| macromolecules | 29.67 |
| ligands | 19.91 |
| solvent | 16.18 |

Statistics for the highest-resolution shell are shown in parentheses.

**References**:

Weiss MS, Hilgenfeld R. 1997. On the use of the merging R factor as a quality indicator for X-ray data. *J Appl Crystallogr* **30**:203–205. doi:10.1107/s0021889897003907

Karplus PA, Diederichs K. 2012. Linking Crystallographic Model and Data Quality. *Science* **336**:1030–1033. doi:10.1126/science.1218231
