# Supplementary material for: Structural evolution of nitrogenase over 3 billion years
Source: eLife. 2025 Sep 11;14:RP105613. doi: 10.7554/eLife.105613 (PMC12425478; doi:10.7554/eLife.105613)
Supplement: Supplementary file 5. [file elife-105613-supp5.docx]

**Supplementary File 5:** Data collection and refinement statistics for Anc1B DDKK

| **Data collection** |  |
| --- | --- |
| Wavelength (Å) | 0.8856 |
| Spherical resolution (Å) | 109.828-1.824 (2.105-1.824) |
| Limiting resolution (Å) along |  |
| a* | 2.805 |
| b* | 2.321 |
| c* | 1.821 |
| Space group | P 2_1_2_1_2_1_ |
| Unit cell | 77.08, 128.98, 209.45, 90.0, 90.0, 90.0 |
| Total reflections | 1198687 (60971) |
| Unique reflections | 91363 (4568) |
| Multiplicity | 13.1 (13.1) |
| Completeness, spherical (%) | 49.1 (7.1) |
| Completeness, ellipsoidal (%) | 94.4 (77.0) |
| Mean I/sigma(I) | 7.5 (2.0) |
| Wilson B-factor | 20.64 |
| R-merge (Weiss and Hilgenfeld,1997) | 0.237 (1.419) |
| R-meas | 0.247 (1.476) |
| R-pim (Weiss and Hilgenfeld,1997) | 0.068 (0.401) |
| CC_1/2_ (Karplus and Diederichs, 2012) | 0.997 (0.727) |
| **Refinement** |  |
| R-work | 0.2063 |
| R-free | 0.2686 |
| RMS(bonds) | 0.075 |
| RMS(angles) | 1.6982 |
| Ramachandran favored (%) | 95.78 |
| Ramachandran allowed (%) | 3.97 |
| Ramachandran outliers (%) | 0.25 |
| Rotamer outliers (%) | 2.46 |
| Clashscore | 6.90 |
| Average B-factor | 25.55 |
| macromolecules | 25.77 |
| ligands | 18.94 |
| solvent | 21.33 |
| Number of TLS groups | 4 |

Statistics for the highest-resolution shell are shown in parentheses.

**References**:

Weiss MS, Hilgenfeld R. 1997. On the use of the merging R factor as a quality indicator for X-ray data. *J Appl Crystallogr* **30**:203–205. doi:10.1107/s0021889897003907

Karplus PA, Diederichs K. 2012. Linking Crystallographic Model and Data Quality. *Science* **336**:1030–1033. doi:10.1126/science.1218231
