# Supplementary material for: Structural evolution of nitrogenase over 3 billion years
Source: eLife. 2025 Sep 11;14:RP105613. doi: 10.7554/eLife.105613 (PMC12425478; doi:10.7554/eLife.105613)
Supplement: Supplementary file 6. [file elife-105613-supp6.docx]

**Supplementary File 6:** Structural features analyzed in the massive nitrogenase structure prediction, with their respective programs.

| Variable | Program | Explanation |
| --- | --- | --- |
| Total area | FreeSASA  (Mitternacht, 2016) | Exposed protein surface, in Å2 |
| Buried area | FreeSASA (Mitternacht 2016) | The sum of the individual chains surfaces minus the protein surface. |
| Polar area | FreeSASA (Mitternacht 2016) |  |
| Polar buried area | FreeSASA (Mitternacht 2016) |  |
| Apolar area | FreeSASA (Mitternacht 2016) |  |
| Apolar buried area | FreeSASA (Mitternacht 2016) |  |
| Intermolecular contacts | Prodigy (Vangone and Bonvin, 2015) | Number of contacts between residues of different chains. |
| Charged-charged contacts | Prodigy (Vangone and Bonvin 2015) | Number of interactions between charged residues of different chains. |
| Charged-polar contacts | Prodigy (Vangone and Bonvin 2015) | Number of interactions between charged residues and polar residues of different chains. |
| Charged-apolar contacts | Prodigy (Vangone and Bonvin 2015) | Number of interactions between charged residues and apolar residues of different chains. |
| Apolar-polar contacts | Prodigy (Vangone and Bonvin 2015) | Number of interactions between apolar residues and polar residues of different chains. |
| Apolar Non-Interacting-Surface | Prodigy (Vangone and Bonvin 2015) | See (Kastritis et al., 2014) |
| Charged Non-Interacting-Surface | Prodigy (Vangone and Bonvin 2015) | See (Kastritis et al. 2014) |
| Binding affinity | Prodigy (Vangone and Bonvin 2015) | Free energy of binding of the complex, in kcal/mol. The lower, the more stable the complex. |
| Gaussian Normal Mode eigenvalues (first five) | Prody (Bakan et al., 2011; Zhang et al., 2021) | Lowest eigenvalues associated with the Gaussian Normal Mode analysis. These values are associated with the frequency of normal mode oscillations (Bauer et al., 2019) |
| Radius of gyration | Prody (Bakan et al. 2011; Zhang et al. 2021) | See (Lobanov et al., 2008) |
| Residue-Residue average shortest path | RING (Clementel et al., 2022) /NetworkX (Hagberg et al., 2008) | Mean length of the shortest path joining two random nodes in a connected network. |
| Residue average clustering | RING (Clementel et al. 2022) /NetworkX (Hagberg et al. 2008) | Number of edges connecting the node and its neighbors between them divided by the number of edges of a complete subgraph involving all its neighbors. |
| Residue network density | RING (Clementel et al. 2022) /NetworkX (Hagberg et al. 2008) | Number of edges divided the number of edges that would be expected if the graph was complete. |
| Residue average degree | RING (Clementel et al. 2022) /NetworkX (Hagberg et al. 2008) | Mean number of neighbors of each residue. |

**REFERENCES**

Bakan A, Meireles LM, Bahar I. 2011. ProDy: Protein Dynamics Inferred from Theory and Experiments. *Bioinformatics* **27**:1575–1577. doi:10.1093/bioinformatics/btr168

Bauer JA, Pavlović J, Bauerová-Hlinková V. 2019. Normal Mode Analysis as a Routine Part of a Structural Investigation. *Molecules* **24**:3293. doi:10.3390/molecules24183293

Clementel D, Conte AD, Monzon AM, Camagni GF, Minervini G, Piovesan D, Tosatto SCE. 2022. RING 3.0: fast generation of probabilistic residue interaction networks from structural ensembles. *Nucleic Acids Res* **50**:W651–W656. doi:10.1093/nar/gkac365

Garcia AK, McShea H, Kolaczkowski B, Kaçar B. 2020. Reconstructing the evolutionary history of nitrogenases: Evidence for ancestral molybdenum‐cofactor utilization. *Geobiology* **18**:394–411. doi:10.1111/gbi.12381

Hagberg AA, Schult DA, Swart PJ. 2008. Exploring Network Structure, Dynamics, and Function using NetworkX 11–15.

Kastritis PL, Rodrigues JPGLM, Folkers GE, Boelens R, Bonvin AMJJ. 2014. Proteins Feel More Than They See: Fine-Tuning of Binding Affinity by Properties of the Non-Interacting Surface. *J Mol Biol* **426**:2632–2652. doi:10.1016/j.jmb.2014.04.017

Lobanov MYu, Bogatyreva NS, Galzitskaya OV. 2008. Radius of gyration as an indicator of protein structure compactness. *Mol Biol* **42**:623–628. doi:10.1134/s0026893308040195

Mitternacht S. 2016. FreeSASA: An open source C library for solvent accessible surface area calculations. *F1000Research* **5**:189. doi:10.12688/f1000research.7931.1

Vangone A, Bonvin AM. 2015. Contacts-based prediction of binding affinity in protein–protein complexes. *Elife* **4**:e07454. doi:10.7554/elife.07454

Zhang S, Krieger JM, Zhang Y, Kaya C, Kaynak B, Mikulska-Ruminska K, Doruker P, Li H, Bahar I. 2021. ProDy 2.0: increased scale and scope after 10 years of protein dynamics modelling with Python. *Bioinformatics* **37**:3657–3659. doi:10.1093/bioinformatics/btab187
